# Electroencephalographic, physiologic and behavioural responses during cervical dislocation euthanasia in turkeys

**DOI:** 10.1186/s12917-019-1885-x

**Published:** 2019-05-07

**Authors:** Elein Hernandez, Fiona James, Stephanie Torrey, Tina Widowski, Karen Schwean-Lardner, Gabrielle Monteith, Patricia V. Turner

**Affiliations:** 1 Depts of Pathobiology, University of Guelph, Guelph, ON N1G 2W1 Canada; 2 Animal Biosciences, University of Guelph, Guelph, ON N1G 2W1 Canada; 3 Clinical Studies, University of Guelph, Guelph, ON N1G 2W1 Canada; 4 College of Agricultural and Bioresources, University of Saskatchewan, Saskatoon, SK S7N 5C9 Canada

**Keywords:** Turkey, Poultry, Euthanasia, Cervical dislocation, Animal welfare, EEG

## Abstract

**Background:**

There is a critical need to develop appropriate on-farm euthanasia methods for poultry species. Euthanasia methods should affect the brain first causing insensibility, followed by cardiorespiratory arrest. Neck or cervical dislocation methods, either manual (CD) or mechanical (MCD), are reported to cause a prolonged time to loss of sensibility and death with inconsistent results upon application, especially MCD methods. However, there is limited information on cervical dislocation in turkeys. The overall objective of this study was to assess the welfare implications of CD and a newly developed MCD device for euthanasia of cull turkeys in comparison with intravenous (IV) pentobarbital sodium (1 mL/4.5 kg), the gold standard euthanasia method. Time to death using electroencephalographic (EEG) and behavioural responses were monitored in eight and eighteen week-old turkeys for five minutes after each euthanasia method application. Spectral analyses of EEG responses and onset of isoelectric EEGs were compared to baseline EEG recordings of birds under anesthesia and behavioural responses were studied among euthanasia treatments. A significant decrease in brain activity frequencies analysis and isoelectric EEG were recorded as time of brain death.

**Results:**

All turkeys euthanized with IV pentobarbital sodium presented a rapid and irreversible decrease in the EEG activity at approximately 30s post-injection with minimal behavioural responses. CD and MCD methods caused EEG responses consistent with brain death at approximately 120 s and 300 s, respectively. Additionally, isoelectric EEGs resulted in all pentobarbital sodium and CD groups, but only in 54 and 88% of the eight and eighteen week-old turkeys in the MCD groups, respectively. There were few clear patterns of behavioural responses after CD and MCD application. However, cessation of body movement and time to isoelectric EEG after CD application were positively correlated.

**Conclusions:**

Use of CD and MCD resulted in a prolonged time to death in both age groups of turkeys. MCD application presents a number of welfare risks based on electroencephalographic and behavioural findings. Intravenous pentobarbital sodium induced rapid brain death, but possesses several on-farm limitations. To develop improvements in cervical dislocation methods, further investigations into combined or alternative methods are required to reduce the prolonged time to insensibility and death.

**Electronic supplementary material:**

The online version of this article (10.1186/s12917-019-1885-x) contains supplementary material, which is available to authorized users.

## Background

On-farm culling of individual sick or injured birds is a routine procedure that has attracted significant attention over the last decade [1]. To be considered humane, killing methods should result in rapid insensibility, followed by irreversible impairment of respiratory function and cardiac arrest [2]. Welfare concerns about different euthanasia methods arise when there is potential induction of pain or distress in injured animals or prolongation of the time to loss of sensibility and onset of death [2, 3].

Animal care guidelines for poultry recommend several methods for on-farm euthanasia including injectable anesthesia agents, and physical methods, such as non-penetrating captive bolt, manual or mechanical cervical dislocation, blunt force trauma, etc. [2, 3]. Intravenous barbiturate overdose (e.g., pentobarbital sodium) is considered the gold standard method of euthanasia method for all species, but effects have not been specifically studied and reported for turkeys [2]. Pentobarbital sodium causes rapid depression of the central nervous system followed by cardiac arrest, although its use is impractical on-farm as it must be administered by a licensed veterinarian with appropriate disposal of injected cadavers [2, 3]. Alternatively, cervical dislocation is a commonly used on-farm euthanasia method in commercial poultry due to its practicality, low cost, ease to use and is only limited by the weight of the animal in most euthanasia guidelines [3, 4]. It can be applied either manually or mechanically with a suitable device that may differ in size, design and application technique, but it should not cause crushing of the cervical vertebrae [2]. The mechanism of action of death by cervical dislocation methods is by cerebral ischemia secondary to stretching and rupture of the major cervical arteries and transection of the spinal cord [1, 5]. However, there are welfare concerns regarding the efficacy of MCD to induce death without pain, prolonged time to loss of sensibility and death following application, and esthetics of technique.

Recently, a new MCD device was developed and is commercially available, but there is no scientific evidence to support its efficacy to kill turkeys. Woolcott et al. (2018) tested the device in poults and reported varied results in dislocation site and vertebral fractures [6]. Additionally, behavioural indicators of insensibility revealed a prolonged duration to loss of sensibility and onset of death after applying MCD compared to CD [6]. Previous encephalographic (EEG) research in turkeys has examined the brain activity following different killing methods by analyzing different quantitative EEG (QEEG) variables derived from power spectrum (spectral edge frequency (F95), median frequency (F50) and total power (Ptot) [7, 8]. Decreases in F95 and F50 together with increases in Ptot values are associated with loss of sensibility and anesthesia, while increases in F50 and F95 with concurrent decreases in Ptot values are associated with death in turkeys [7]. Similarly, other EEG data can be obtained to study sensibility vs. insensibility by interpretation of changes in the background activity (“waveforms” or frequency bands) of the brain (e.g. delta, theta, alpha, and beta) [9]. While delta and theta are low frequency waves associated with sleep or insensible states, alpha and beta are high frequency, low amplitude waves commonly observed in awake states. Frequency band analyses have been less commonly employed in species other than humans to date. However, this analysis technique has been validated previously in poultry [9] and several other research groups have used frequency band analyses in sheep, dogs, and alligators [10–12].

Most investigations of turkey euthanasia are based on behavioural and physiologic assessment or histopathology evaluations [6, 13–15]. However, there is scarce information on turkey EEGs and the relationship between physiologic, behavioural and EEG assessments after the application of cervical dislocation in turkeys. Further research on comparisons of behavioural, physiologic and EEG changes with different states of sensibility is needed to understand when death occurs in turkeys. When there are concerns regarding potential pain or welfare outcomes for untested killing techniques, a minimal anesthesia model can be used to protect animal welfare [7, 16, 17]. However, interpretation of results should be conducted cautiously, because the results may be impacted by the anesthetic agent [6, 7, 16] and this method also limits evaluation of the transition between sensible to insensible states [16].

The objective of this study was to assess the welfare implications of manual cervical dislocation and mechanical cervical dislocation using the Koechner Euthanasia Device® for euthanasia of cull turkeys and comparing with intravenous pentobarbital sodium (1 mL/4.5 kg) using a minimal anesthesia model. This was studied using EEG, reflex and behavioural responses in eight and eighteen week-old turkeys that were lightly anesthetized with isoflurane/O_2_. Two secondary objectives were to determine the effect of the inhalant anesthesia on behavioural and EEG responses using a minimal anesthesia model, and to compare different quantitative EEG variables in response to different euthanasia methods. We hypothesized that there would be a different time to brain death between euthanasia methods.

## Results

### Analyses of parameters associated with insensibility and death

The incidence of various behavioural and physiologic parameters occurring after euthanasia method application is presented in Table 1. The frequencies of tonic and/or clonic convulsions, gasping, loss of nictitating membrane reflex, loss of pupillary light reflex, and cardiac arrest were affected by the euthanasia method when comparing CD to pentobarbital sodium and MCD to pentobarbital sodium in 8 week-old (w.o.) turkeys. The only frequencies affected by euthanasia method in 18 w.o. turkeys were clonic convulsions, gasping, and loss of nictitating membrane reflex. More turkeys were observed with tonic and clonic convulsions in 8 w.o. birds euthanized with CD and MCD vs. pentobarbital sodium (clonic: 63% vs. 0%, *p =* 0.03; tonic: 88% vs. 0%, *p =* 0.0005; and clonic: 92% vs. 0%, *p =* 0.0004; tonic: 73% vs. 0%, *p =* 0.01, respectively). Similarly, more 18 w.o. birds experienced clonic and tonic convulsions following MCD compared to pentobarbital sodium (clonic: 100% vs. 0%, *p =* 0.0003; tonic: 67% vs. 0%, *p =* 0.3, respectively). There was an effect of method on the number of birds demonstrating negative nictitating membrane reflexes following MCD application in both age groups of turkeys, compared to pentobarbital sodium (17% vs. 100%, *p =* 0.001; 40% vs. 100%, *p =* 0.01, respectively). In contrast, there was only a significant difference in the number of birds with loss of pupillary light reflex in 8 w.o. turkeys after MCD compared with pentobarbital sodium (MCD vs. pentobarbital: 17% vs. 100%, *p =* 0.001; CD vs. pentobarbital: 60% vs. 100%, *p =* 0.3, respectively). Fewer 8 w.o. turkeys presented with cardiac arrest after CD and MCD application compared to pentobarbital sodium (1% vs. 100%, *p =* 0.005, 8% vs. 100%, *p =* 0.0004, respectively).

**Table 1:** Incidence (and percentage) of 8 and 18 w.o. turkeys presenting with various behavioural and physiologic parameters and isoelectric EEG in the 5 min following euthanasia method application

Parameter	8 w.o.			18 w.o.	
	Pentobarbital sodium (*n* = 6)	CD (*n* = 8)	MCD (*n* = 12)	Pentobarbital sodium (*n* = 5)^1^	MCD (*n* = 10)
Clonic convulsions	0^a^ (0)	5^b^ (63)	11^b^ (92)	0^c^ (0)	10^d^(100)
Tonic convulsions	0^a^ (0)	7^b^ (88)	8^b^ (73)	0 (0)	6 (67)
Feather erection	4 (67)	5 (63)	8 (73)	5 (100)	8 (80)
Gasping	0^a^ (0)	5^b^ (63)	12^b^ (100)	0^c^ (0)	10^d^ (100)
Loss of nictitating membrane	6^a^ (100)	5^a^ (63)	2^b^ (17)	5^c^ (100)	4^d^ (40)
Loss of pupillary light reflex	6^a^ (100)	4^a^ (50)	2^b^ (17)	5 (100)	6 (60)
Cardiac arrest	6^a^ (100)	1^b^ (13)	1^b^ (8)	−^2^	
Isoelectric EEG	6 (100)	8 (100)	6 (50)	4 (100)	8 (80)

^1^One EEG recording was not available for evaluation

^2^ Data not collected due to sampling limitations

^a,b^ Indicates differences from chance alone within 8 w.o. turkeys for CD or MCD compared to pentobarbital sodium based on 2 × 2 contingency tables

^c,d^ Indicates differences from chance alone within 18 w.o. turkeys for CD or MCD compared to pentobarbital sodium based on 2 × 2 contingency tables

Statistical analyses demonstrated no overall effect of age × treatment interaction on the latencies to tonic convulsions, gasping, feather erection, and time to last movement in the turkeys. However, there was an effect of age on the latency to clonic convulsions and euthanasia method as well on the latency to feather erection and time to last movement (Table 2). There was no effect of age on the duration of the responses following euthanasia device application (Table 3). The latency to onset of clonic convulsions was affected by age with 18 w.o. turkeys demonstrating a shorter latency compared to 8 w.o. birds (11 ± 20s vs. 136 ± 19 s; *p* = 0.0002, Table 2). Time to last movement and onset of feather erection showed an effect of euthanasia method, with birds euthanized with pentobarbital sodium presenting a shorter onset compared to CD and MCD (time to last movement: 1 ± 1 s vs. 231 ± 27 s vs. 220 ± 16 s; *p* = < 0.0001; feather erection: 47 ± 30s vs. 150 ± 35 s vs. 207 ± 26 s; *p* = 0.003, respectively). No age effect (*p* = 0.6, *p* = 0.4) or method × age interaction (*p* = 0.6, *p* = 0.4) were observed for time to last movement and feather erection, respectively.

**Table 2:** Mean ± SE latency (s) to onset of physiologic and behavioural parameters following euthanasia method application in 8 and 18 w.o. turkeys

Age (weeks)	Method			*p* value		
	Pentobarbital sodium	MCD	CD	Method^1^	Age^2^	Method x age
Clonic convulsions						
8	–	136 ± 19^a^	78 ± 31	0.09	0.0002	–
18	–	11 ± 20^b^				
Mean		73 ± 14	78 ± 31			
Tonic convulsions						
8	–	205 ± 26	153 ± 30	0.2	0.2	–
18	–	150 ± 30				
Mean		178 ± 20	153 ± 30			
Time to last movement						
8	1 ± 1	237 ± 22	231 ± 27	< 0.0001	0.6	0.6
18	1 ± 1	204 ± 24				
Mean	1 ± 1^c^	220 ± 16^d^	231 ± 27^d^			
Gasping						
8	–	4 ± 4	4 ± 3	0.5	0.06	–
18	–	15 ± 5				
Mean		10 ± 3	4 ± 3			
Feather erection						
8	50 ± 43	171 ± 30	150 ± 35	0.003	0.4	0.4
18	45 ± 43	241 ± 43				
Mean	47 ± 30^c^	207 ± 26^d^	150 ± 35^d^			
Isoelectric EEG						
8	18 ± 36	278 ± 36	239 ± 31	< 0.0001	0.5	0.4
18	26 ± 40	218 ± 31				
Mean	22 ± 27 ^c^	248 ± 24 ^d^	239 ± 31^d^			

^1^ The effect of method compares pentobarbital sodium vs. mechanical cervical dislocation (MCD) vs. manual cervical dislocation (CD)

^2^The effect of age compares the response of pentobarbital sodium and MCD between 8 and 18 w.o. turkeys

^a,b^Indicates differences observed within column (difference within age)

^c,d^Indicates differences observed within a row (difference within method)

**Table 3:** Mean ± SE duration (s) of behavioural and physiologic responses for each euthanasia method in 8 and 18 w.o. turkeys

Parameters	Method^1^			Analysis	
	Pentobarbital sodium	CD	MCD	F	*p* value
Clonic convulsions	–	20 ± 17^a^	62 ± 10^b^	6.8	0.003
Tonic convulsions	–	50 ± 7^a^	23 ± 4^b^	13.6	< 0.0001
Motionless	254 ± 22	214 ± 26	202 ± 16	1.9	0.2
Gasping	–	45 ± 22^a^	253 ± 13^b^	71.3	< 0.0001
Feather erection	38 ± 10	48.5 ± 12	19 ± 7	2.7	0.1

^1^The effect of method compares pentobarbital sodium vs. mechanical cervical dislocation (MCD) vs. manual cervical dislocation (CD)

^a,b^ Indicated differences observed within row (difference within method)

Groups that did not present a particular response were scored as missing values, “-”.

Turkeys euthanized by CD exhibited a shorter duration of clonic convulsions compared to MCD (20 ± 17 s vs. 62 ± 10s; *p* = 0.003). By contrast, tonic convulsions lasted longer in the CD group compared to MCD (50 ± 7 s vs. 23 ± 4 s; *p* = < 0.0001). Birds euthanized by pentobarbital sodium overdose had a longer duration of time spent motionless compared to CD and MCD (254 ± 22 s vs. 214 ± 26 s and. 202 ± 16 s; *p* = 0.2). Gasping was only observed in birds euthanized by CD and MCD, and turkeys euthanized by MCD exhibited a longer duration of gasping compared to those euthanized by CD (253 ± 13 s vs. 45 ± 22 s; *p* = < 0.0001).

Groups that did not present the responses were scored as missing values “-”.

### EEG analyses

EEG recordings were successfully collected from instrumented birds with the exception of one EEG from an 18 w.o. bird in the pentobarbital sodium group. The EEG recording was acquired but not saved properly for later analysis due to unexpected technical issues after the recording concluded. Remaining data sets were complete. Statistical analyses demonstrated no overall effect of trial or age × treatment interaction on any EEG frequency bands and spectral frequencies (Tables 4 and 5), except for F50 in birds euthanized with pentobarbital sodium (Table 6).

**Table 4:** Summary effects of euthanasia method on EEG frequency bands in 8 and 18 w.o. turkeys

	Delta (0–4 Hz)		Theta (4–8 Hz)		Alpha (8–13 Hz)		Beta (14-30 Hz)	
Effect	*F*	Pr > *F*	*F*	Pr > *F*	*F*	Pr > *F*	*F*	Pr > *F*
Treatment	10	0.0003	118.5	< 0.0001	21.9	< 0.0001	18.2	< 0.0001
Time	3.3	<.0001	2.5	0.0003	6.5	< 0.0001	4.1	< 0.0001
Treatment x Time	1.4	0.05	2.1	0.0033	1.6	0.02	1.9	0.002

**Table 5:** Summary effects of euthanasia method on EEG spectral frequencies and Ptot in 8 and 18 w.o. turkeys

	F50		F95		PTOT	
Effect	*F*	Pr > *F*	*F*	Pr > *F*	*F*	Pr > *F*
Treatment	35.2	< 0.0001	17.9	< 0.0001	30.2	< 0.0001
Time	2.1	0.007	4.1	< 0.0001	5.6	< 0.0001
Age x Time	2	0.009	–	–	–	–
Treatment x Time	2.7	0.0004	1.2	0.2	2	0.0004
Age x Treatment x Time	2.3	0.002	–	–	–	–

**Table 6:** Comparison of the mean (±SE) F50 after administration of pentobarbital injection

	F50, Hz	
Time(s)	8 w.o.	18 w.o.
Baseline	5.1 (1.0)	4.0 (1.1)
15	5.7 (1.0)	4.9 (1.1)
30	7.0 (1.0)*	9.7 (1.1)*
45	6.7 (1.0)	6.7 (1.1)
60	9.7 (1.0)	7.6 (1.1)*
75	9.3 (1.0)	7.1 (1.1)*
90	8 (1.0)	5.3 (1.1)*
105	6 (1.0)^*^	10.1 (1.1)
120	11.3 (1.0)	7.7 (1.2)*
135	5.7 (1.0)	8.0 (1.1)
150	6.3 (1.0)^*^	10.4 (1.1)
165	6.7 (1.0)^*^	8.3 (1.1)
180	6.3 (1.0)	5.6 (1.1)
195	6.3 (1.0)^*^	8.5 (1.1)
210	7.1 (1.1)	7.0 (1.2)
225	6.0 (1.0)^*^	9.7 (1.4)
240	6.0 (1.1)	7.1 (1.4)
255	7.2 (1.2)	9.0 (1.7)
270	7.7 (1.2)	5.1 (2.4)
285	6.2 (1.4)	5.7 (2.4)
300	5.5 (1.5)	8.5 (2.6)

Baseline results represent the mean F50 for the 4 s immediately before euthanasia.

* indicate times at which 8 and 18 w.o. means differed between ages at a particular time point (*p* < 0.05)

**Fig. 1:**
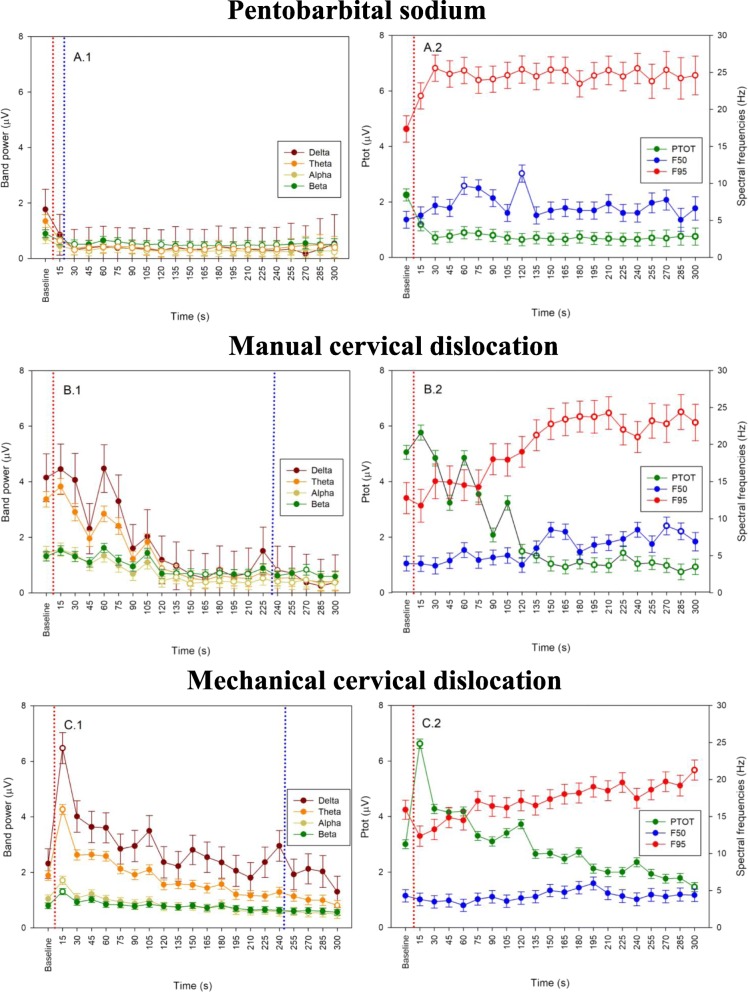
EEG analyses following euthanasia application in 8 and 18 w.o. turkeys. Shown are the time course to brain death following administration of pentobarbital sodium IV (**a**), manual cervical dislocation (**b**), and mechanical cervical dislocation (**c**) of mean (±SE) band power (A.1, B.1, C.1) and mean (±SE) Ptot with spectral variables (F50 and F95) (A.2, B.2, C.2) from baseline to 5 min endpoint (300 s). Data are shown as mean (±SE) values for consecutive 15 s intervals after euthanasia application. Baseline values represent the mean of four 15 s intervals immediately preceding euthanasia. Open symbols indicate post-euthanasia time points at which mean differed from baseline within treatment (adjusted *p* < 0.05). The dotted red line represents time of euthanasia method application and dotted blue line represents onset of isoelectric EEG

Mean EEG pattern changes over time for spectral frequencies and latency of isoelectric EEGs are shown in Fig. 1. All birds maintained a pattern of increased Ptot, delta and theta frequencies with suppressed alpha and beta frequency bands during baseline recordings and at the time of application of the euthanasia method that represent insensibility secondary to anesthesia. However, the frequency patterns generally changed significantly following method application lasting up to the estimated time to brain breath. Burst suppression patterns, defined as high voltage activity alternating with periods of no activity (isoelectric), were noted in eleven birds (8 w.o., *n* = 9; 18 w.o., *n* = 2 birds) immediately after isoflurane induction and these patterns were generally maintained during light anesthesia (1.5% isoflurane/O_2_) baseline recordings. There was a significant euthanasia method × time effect on frequency bands (alpha, beta, theta, delta), F50, and Ptot, in addition to a significant time effect on F95 (Tables 4, 5, 6). There was no age effect in spectral frequencies, Ptot and frequency bands regardless of whether turkeys were euthanized with MCD or CD (*p* > 0.05, Tables 4 and 5). By contrast, there was an age effect in turkeys euthanized with pentobarbital sodium, demonstrated as an increase in F50 at different time points between ages (Table 6). Mean theta and Ptot demonstrated a decrease from 15 to 300 s in response to pentobarbital injection (adjusted *p* < 0.05, see [Additional file 1]). For alpha, beta, and F95, there was a significant decrease over the period of 30s to 300 s after pentobarbital sodium administration (adjusted *p* < 0.05, Fig. 1-A.1). These changes occurred approximately at the same time as the visual determination of isoelectric EEG and a corresponding significant increase was seen in F90 and F50 at 30s and 60s, respectively, after pentobarbital injection (Fig. 1-A.2).

For CD, theta, alpha, beta, and Ptot decreased significantly between 120 and 300 s (Fig. 1-B.1, [see Additional file 2]), with changes in delta and F95 from 135 s until 300 s (*p* < 0.05, Fig. 1-B.2). All birds demonstrated isoelectric EEGs at approximately 239 ± 31 s after CD application. Changes in mean alpha frequency bands were observed from 210 s until the 5-min endpoint after MCD application (*p* < 0.05; Fig. 1-C.1, [see Additional file 3]). Mean theta, F95 and Ptot were significantly different compared to baseline at 300 s (*p* < 0.05, Fig. 1-C.2), with isoelectric EEG noted at approximately the same time (at 248 ± 24 s, Fig. 1-C.1). There was no method effect on the number of birds presenting with isoelectric EEG after MCD in 8 and 18 w.o. turkeys, compared to pentobarbital sodium (MCD vs. pentobarbital sodium: 50% vs. 100%, *p =* 0.05; MCD vs. pentobarbital sodium: 80% vs. 100%, *p =* 0.5, respectively).

### Relationship between EEGs and insensibility and brain death responses

Changes in EEG spectral analyses (background activity [alpha, beta, theta and delta] and spectral frequencies with Ptot) were highly associated with observed isoelectric points across treatments. Isoelectric EEG and spectral frequency changes associated with brain death occurred within 15 s of pentobarbital sodium administration, but they were not seen until 120–135 s for CD, and 300 s for MCD with isoelectric changes observed at 240 s for both dislocation methods. After pentobarbital sodium injection, turkeys exhibited minimal movements or behavioural and physiologic responses with rapid onset of brain death and isoelectric EEGs. CD and MCD treatments caused a rapid onset of gasping followed by clonic-tonic convulsions and feather erection prior to onset of isoelectric EEG - all at different time points across treatments. Cessation of body movements, measured as time to last movement, was positively correlated with onset of isoelectric EEG (*r* = 0.8; *p* = 0.03) in birds euthanized with CD, but not for MCD or pentobarbital sodium (*r* = 0.2; *p* = 0.6 & *r* = 0.2 *p* = 0.7, respectively). In the latter case, this was likely because pentobarbital sodium did not induce much in the way of body movement prior to death. Cardiac arrest was observed in all birds euthanized with pentobarbital sodium and in two birds at 5 min after CD and MCD application (8 w.o.: CD *n* = 1; MCD *n* = 1). In contrast, feather erection was observed in > 50% of all euthanized turkeys, regardless of euthanasia method.

There were significant age × time, treatment × time, and age × time × method interactions in F50 after pentobarbital sodium injection (*p* = 0.009, *p* = 0.0004; *p* = 0.002, respectively, Table 5). The significant age-related changes were present between 30 and 225 s (Table 6). However, these changes were not associated with other significant differences in EEG variables or other observed behavioural or physiologic parameters among age groups.

CD and MCD caused sharp increases and peaks in Ptot and delta waves after application, although the timing and magnitude were different between treatments. For CD, the peaks occurred at 60s (delta, adjusted *p* = 0.7; Ptot, adjusted *p* = 0.9) and 105 s (delta, adjusted *p* = 0.2; Ptot, adjusted *p* = 0.2) after application, with no relationship to other insensibility parameters except for mean latency of clonic convulsions. For MCD, the peak (delta, adjusted *p* = 0.002; Ptot, adjusted *p* = 0.002) occurred 15 s after application with concurrent onset of gasping in both age groups and clonic convulsions in 18 w.o. turkeys.

## Discussion

This study compared the differences in behavioural and physiologic responses to EEG activity in eight and eighteen w.o. lightly anesthetized turkeys euthanized by pentobarbital sodium overdose, CD or MCD. The most important finding was that the CD and MCD methods resulted in a prolonged time (120–300 s) to brain death based on EEG parameters compared to pentobarbital sodium. As anticipated, our results indicated that IV pentobarbital sodium induced a rapid, smooth and uneventful depression of the central nervous system followed by death. These findings confirm the use of IV pentobarbital sodium as the gold standard euthanasia method in mature and immature turkeys by causing immediate signs of insensibility and death within 60s or less after drug administration. Conversely, birds euthanized either by CD or MCD demonstrated variable and prolonged latency to brain death based on behavioural and physiologic responses and EEG changes.

The welfare implications of each euthanasia method were assessed based on the efficacy of the method to induce rapid brain death (based on EEG changes) as well as by examining the relationship between EEG changes and occurrence of common physiologic and behavioural responses of insensibility and death in turkeys [6, 7]. Despite the specific weight-based design of the MCD tool (i.e., KED-C and KED-M for this study), this method was ineffective for killing all birds. This finding also reinforced the value of our conservative approach for using light inhalant anesthesia for protecting the welfare of the birds. Isoelectric EEGs were only observed in approximately 50 and 80% of the eight- and eighteen w.o. turkeys, respectively, after MCD application with EEG activity significantly depressed (i.e., decreased delta, theta and Ptot compared to baseline) at about 300 s after application. In contrast, all turkeys euthanized either with pentobarbital sodium or CD developed an isoelectric EEG with decreased EEG activity at approximately 15–30s and 120 s, respectively. In addition, CD consistently resulted in a shorter mean latency to isoelectric EEG onset compared with MCD. Similar findings were reported by Gregory and Wotton (1990); they described a shorter time to loss of visual evoked potential (an indicator of brain death) in chickens euthanized with a method that stretched the neck compared to MCD using a commercial set of pliers [5]. The use of raw inspection of the EEG to determine isoelectric EEG as well as assessment of frequency bands and other spectral frequency analyses, provided more in-depth understanding of brain activity changes when transitioning from anesthetic-induced insensibility to death following application of each method.

In this study, there was no clear pattern of changes between EEG and behavioural measures across ages and methods, as described by Martin (2015) [18]. The poor correlation between behavioural and physiologic responses likely results from the different mechanism of action for inducing death between chemical and physical methods as well as differences in the efficacy of each method. Feather erection has been suggested as an indicator of cardiac arrest [19]. However, there was no clear association between cardiac arrest and feather erection since feather erection was observed in the majority of birds with a heartbeat after CD and MCD application. Similarly, cardiac arrest was observed in all turkeys after pentobarbital sodium injection, but feather erection was not observed in all birds. Cardiac arrest following a barbiturate overdose occurs secondary to direct physiologic inhibition of cardiac motility as well as significant central respiratory depression resulting in hypoxia, anoxia and brain death [20]. In contrast, CD and MCD induce direct trauma to the spinal cord with disruption of the spinal and cerebral blood supplies [5, 16] and these methods do not directly impact cardiac function. Failure to induce cardiac arrest in turkeys after MCD application with the KED-S was also described by Woolcott [6], but feather erection evaluated in that study.

Cessation of body movements and time to brain death as assessed by onset of an isoelectric EEG were the only parameters and EEG events closely correlated after CD application (*r* = 0.8; *p* = 0.03). This is similar to previous investigations studying depopulation methods in chickens [21] and has been inferred in previous turkey studies [13, 15]. The incidence of clonic-tonic convulsions in the current study could have been affected by the inhalant anesthesia [13]. However, other studies in poultry suggest that convulsions are not reliable indices of insensibility or brain death [13, 16]. In the current study, using the recommended MCD tool sizes for each age group, five of six turkeys (four and two turkeys from the eight and eighteen w.o. groups, respectively) that did not show an isoelectric EEG after MCD application exhibited other indicators of insensibility including clonic-tonic convulsions or loss of brainstem reflexes at different time points. However, one of the 22 birds investigated with this method presented no laboured breathing, clonic-tonic activity or isoelectric EEG before the five-minute endpoint, with a persistent eye reflex after the device application. It is likely that this bird remained insensible but presented a prolonged time to brain death secondary to a failed device application. Similarly, not all turkeys developed clonic-tonic convulsions after CD application, but all did develop an isoelectric EEG. Thus, this work confirms that demonstration of clonic-tonic convulsions cannot be used reliably to predict brain death in all turkeys after application of physical euthanasia methods. Presence of eye reflexes are considered conservative indicators of insensibility and death [6, 7, 22]; however, some eye reflexes can occasionally be observed in birds under light anesthesia and eye reflex information should be combined with other insensibility indicators when making decisions about brain death [23]. Our findings reinforce the inconsistency and inefficiency of the MCD device for inducing rapid death based on EEG data together with reflex and physiologic and behavioural findings. This is in agreement with other studies describing sub-optimal and inconsistent kill performance of various other cervical dislocation killing devices in poultry [1, 6, 13].

This study also aimed to compare different QEEG variables for validation of different types of EEG analyses in turkeys. The EEG data presented complement previously reported results of the effects of inhalant anesthesia, pentobarbital sodium administration, and cervical dislocation in birds and other species [7, 24–26]. However, species-specific differences and variations in data collection methods and analyses do not permit direct comparisons of values between studies. Overall EEG activity during baseline recording was composed of high-amplitude, low frequency activity quantified by delta and theta power bands similar to studies reported in both mammals and birds [7, 10, 25–27]. For F50 and F95, a similar pattern of increased Ptot and decreased F50 and F95 during anesthesia has been reported [7, 28]. In this study, all turkeys presented with similar values in F50 related to insensibility during the baseline period and after euthanasia application (non-responsive state: F50 < 12.7 Hz; general anesthetic plane: F50 < 6.8 Hz) [29]. Pentobarbital sodium caused an immediate and significant transition from anesthetic-induced insensibility to death, characterized by an overall decrease in mean values of band powers and total power with a mild to moderate increase in mean spectral edge (F95) and median (F50) frequencies. Sandercock et al. (2014) reported similar changes in F95 and F50 for baseline EEG and a characteristic decreased EEG activity, reflected in Ptot, after pentobarbital sodium administration [7]. Differences between age groups in F50 starting at 30s after injection were not considered relevant and were likely secondary to individual variation or age-dependent effect [30]. Similarly, changes observed after onset of transitional or isoelectric EEG are not likely representative of accurate brain activity but external noise [25]. For MCD, a significant and gradual increase in F95 was observed at 225 s with an overall decrease in brain wave activity at approximately at 300 s, suggesting time to loss of cerebral function. This differed from turkeys euthanized with CD, which instead demonstrated a sustained but not significant increase in all frequency bands followed by a significant decrease from 135 to 300 s associated with a transient increase in F95, indicative of cessation of brain activity. In human encephalopathies, slowing of EEG activity with increased mean theta and delta powers for 1 to 2 min are described following acute brain trauma or chronic spinal cord injury [31, 32]. Therefore, it is likely the sustained increases in delta and theta are secondary to the external mechanical insult to the central nervous system caused by the cervical dislocation methods and validates the use of frequency bands analysis in turkeys for EEG These changes could be also attributed to severe hypoglycemia or electrolyte alterations that are the result of the varied degree of dislocation induced by these physical methods [33, 34].

The acute increase in Ptot observed after CD and MCD has been described as loss of functional cerebral activity in chickens undergoing CD [16]. However, studies of different killing methods in mammals comparing anesthetized and non-anesthetized animals have attributed the increase in Ptot to noxious stimuli [26, 35, 36]. In our study, there was no shift in the EEG frequency bands from delta and theta to alpha and beta when Ptot increased that could be associated with regaining sensibility or noxious stimulation [27]. Similarly, there was no simultaneous increase in F50 and F95 that could be interpreted as a noxious stimulus response as suggested by some authors [25, 27, 37]. Changes in the EEG were interpreted as an overall arousal of theta and delta waves causing an increase in Ptot [24, 25]. F50 and F95 did not provide significant information on transition from insensibility to death, as expected [7]. The possibility that isoflurane caused more pronounced CNS depression compared to other anesthetic agents used in previous studies in chickens cannot be discounted [25, 27, 38]. In addition, pain evaluation in the chickens and turkeys using EEG is poorly understood given the anatomical differences between the mammalian and avian brain [38]. It is unknown if non-anesthetized turkeys euthanized with MCD would pain or present shifts towards sensitive states based on potentially painful lesions (e.g. vertebral fractures, skin lacerations and trachea collapse) associated with application of this device [6].

From a welfare point of view, time to onset of insensibility is critical [39], but the use of a minimal anesthesia model in EEG studies is valid if there are welfare concerns [38, 40, 41]. Given the unknown outcome of the KED devices, the turkeys were euthanized under light isoflurane anesthesia in this study. Inhalant anesthesia induces a dose-dependent, reversible depression of the CNS, but there is little information available on simultaneous EEG and behavioural responses or the comparison of EEG data on anesthetized vs. non-anesthetized birds [5, 38]. Recent studies in rats have reported short delays in EEG responses in anesthetized rats compared to non-anesthetized rats [36, 42]. Sandercock and colleagues (2014) described the EEG and eye reflex responses in awake, sedated, anesthetized, and dead 11-week-old turkeys, but the authors did not evaluate the anesthesia effect on the timing of events [7]. Our findings suggest that isoflurane had minimal effects on the outcome of the experiments since we observed similar responses at similar times as reported by previous authors investigating common insensibility indicators used on farm in turkey production [6, 22]. Additionally, and in agreement with previous anesthesia studies in chickens and turkeys [7, 26, 43], burst suppression patterns were observed during baseline recordings in the two age groups turkeys. Burst suppression has been suggested to occur at a deeper surgical depth of anesthesia in other species. However, turkeys could have a species-specific anesthetic sensitivity [7]. Sandercock and colleagues (2014) briefly described burst suppression activity and respiratory depression in ten-week old turkeys and mature chickens anesthetized with sevoflurane but did not described in detail the incidence or burst suppression pattern between differences species [7].

In the current study, eye reflexes were evaluated based on presence or absence within 5 min to support the analysis of time to brain death based on EEG findings. CD and MCD were not effective in abolishing nictitating membrane and pupillary light reflexes in 100% of the birds. This was also reported in previous research conducted in turkeys in which eye reflexes persisted immediately after CD and MCD application [6, 13]. In addition, Woolcott (2018) reported more turkeys without immediate loss of nictitating membrane reflex after MCD using an adapted mechanical device (KED-S, size small) compared to CD, but no difference in number of birds with loss of pupillary light reflexes in 3 w.o. anesthetized turkeys [6]. However, a different sedation and anesthesia protocol was used in that study [6]. Additional factors that could have affected the analysis and interpretation of the results were bird size and age, use of a secondary euthanasia method, rearing conditions of the birds, and sex. The bird size is directly correlated with the age and only the recommended methods could be applied according to the body weight. The MCD performance was not likely affected by these factors since recommended devices according to body weight were used. Radiographic evidence or dissections of the cadavers were not conducted in this study since the secondary method could have affected the interpretation of the results [14], but were done in a pilot study to confirm correct application of the methods (data not shown). Recent studies confirm the poor efficacy and welfare issues of applying the KED in chickens and turkeys [44, 45]. A study assessing radiograph scoring of dislocation site of turkeys of the same age groups revealed displacement between the skull and first vertebrae and first and second vertebrae in 9/13 and 4/13 8 w.o. turkeys, respectively. In 18 w.o toms, cervical displacement was observed between the skull and first vertebrae and first and second vertebrae in 6/8 and 2/8 turkeys, respectively [44, Widowski, TM unpublished). CD could only be assessed in 8 w.o. birds due to body size limitations and its application could have been affected by inherent anatomic features (neck with stronger musculature compared to chickens). However, the shorter latency to isoelectric EEG suggests a better outcome of the method for inducing death compared with MCD in 8 w.o. turkeys. Rearing conditions and inclusion criteria of the birds were different but this likely did not affect EEG and behavioural responses, since the birds were alert and responsive and only presented signs of illness that affect production parameters (e.g., hock lesions and pendulous crop). The sex of the birds differed among age groups but was due to the availability of animals at the time of the experiment.

## Conclusion

In conclusion, this study is the first to quantitatively examine the relationship between pentobarbital sodium and two cervical dislocation methods with different EEG variables in turkeys. In terms of animal welfare, time to insensibility is more relevant than time to death but could not be evaluated given the use of a minimal anesthesia model to ensure the welfare of the animals undergoing euthanasia. However, time of death also provides significant information on the efficacy and humanness of an euthanasia method. Application of the mechanical euthanasia devices (KED-C and KED-L) resulted in highly inconsistent and prolonged times to death compared to manual CD or IV pentobarbital sodium without clear behavioural or physiologic responses to determine brain death. There are still several welfare concerns with the use of CD including prolonged time to brain death, but time to cessation of movements is highly associated with time to death. Further refinement and training optimization might improve the performance and reliability of the MCD device in heavier turkeys. These data provide quantitative information for future recommendation on poultry euthanasia.

## Methods

### Animals and study approval

Twenty-six immature female turkeys (8 w.o., mean ± S.E. body weight = 2.5 ± 0.6 kg) were sourced from one commercial farm near Guelph, ON, Canada, in addition to 15 mature toms (18 w.o., mean ± S.E. body weight = 19.4 ± 1.2 kg) from the University of Guelph (Table 7). The owner of the private flock consented to participate in the trial and the study was conducted in a procedure room on- farm. All birds were housed in same-sex flocks and were selected for culling based on farm animal care protocols. Inclusion criteria were clinically injured 8 w.o. turkeys that were unlikely to recover or birds designated as unfit for transport. Specifically, the female birds included in this study presented with hock lesions or a pendulous crop whereas the mature toms had reached their experimental endpoint at the university research station. All animal work was approved by the University of Guelph Animal Care Committee (AUP 3321), which holds a Good Animal Practice certificate issued by the Canadian Council on Animal Care.

**Table 7:** Distribution of treatments and sample size for each euthanasia method

Age (weeks), sex	Weight (kg)	Method	Sample size
8, female	2.0 ± 0.5	Pentobarbital sodium	6
8, female	2.8 ± 0.8	CD	8
8, female	2.8 ± 0.7	MCD	12
18, male	19.0 ± 1.3	Pentobarbital sodium	5
18, male	19.5 ± 1.1	MCD	10

### Instrumentation of EEG electrodes

Birds were brought to the procedure room and kept in a pen for 2–4 h prior to study start. Each turkey was individually induced by facemask with isoflurane (5%, Isoflo, Abbot Laboratories, IL, USA) in 100% oxygen. Indicators of sensibility, such as loss of leg tone (i.e. tension in lifting the leg) and toe pinch (i.e. withdrawal of the foot in response to pressure to the toes), were assessed for onset of insensibility [19]. Birds were maintained in a light anesthetic state until the euthanasia method was applied, at which point the isoflurane and oxygen were turned off. Electrode placement and analyses were modified from Eberle et al. (2018) and James et al. (2017) [11, 46]. Briefly, four 25-gauge needle EEG subdermal wire electrodes (Ives EEG Solutions, MA, USA) were placed 0.5 to 1 cm dorsolateral over the right and left cerebrum (corresponding to rostral and caudal halves of each hemisphere). The electrodes were inserted subcutaneously (SC) overlying the frontal bone and above the cerebrum using the midpoint area between the eyes and the ear openings as anatomic landmarks [13]. A reference electrode was inserted SC overlying the anterior frontal bone and an indifferent (neutral) electrode was inserted SC overlying the occipital bone. All electrode tips were secured onto the head with surgical tape. The electrode adapter was plugged into the colour-coded lead wires and these wires were secured to the facemask and rebreathing tube with surgical tape.

### Experimental design

All birds were randomly allocated to one of three treatments on the day of each trial using a random number generator (random.org): pentobarbital sodium overdose, CD and MCD (Table 7). CD was not applied to the older turkeys as per weight restrictions in Canadian animal care guidelines for poultry [3]. Birds were euthanized by an experienced operator over eight experimental trials, with no more than 6 birds being tested either with CD or MCD per day to minimize operator fatigue. Humane endpoints included inability to collect a valid EEG signal, prolonged laboured breathing, and inability to detect cervical damage through palpation. When any of these conditions occurred a second euthanasia method was applied (i.e., manual cervical dislocation in 8 w.o. birds and non-penetrating captive bolt in 18 w.o. birds).

### Baseline EEG recording

Lightly anesthetized birds were placed in a sternal position with simultaneous collection of video and EEG recordings for 5 min to obtain baseline behaviours and brain activity. Birds were also video and EEG recorded for 5 min beginning immediately after the euthanasia method was applied.

### Euthanasia methods and procedures

Barbiturate overdose consisted of pentobarbital sodium (Euthansol, Intervet Canada Corp., Merck Animal Health, Canada, 340 mg/ml) dosed at 1.5 mL/kg and administered intravenously as a bolus into the brachial vein within 5–10 s after needle placement. MCD was applied by using commercially available non-penetrating forceps, Koechner Euthanasia Device or KED, designed by Koechner MFG. Co, Inc. [47]. Two variations of the KED were assessed based on the weight classes of the birds, KED-C and KED-T. KED-C (size medium) was designed for birds up to 13.6 kg and consists of a 69 cm handle with gear. KED-T (size large) is recommended for use on turkeys up to 20.4 kg and consists of a 102 cm handle with gear [6, 47]. The KED-C was used for 8 w.o. birds and the KED-T was used for 18 w.o. turkeys. For both KED tools, the birds were restrained by the legs with the neck stretched and maintained in sternal position by one operator while a trained operator applied the tool by closing the handles after previously placing the blades behind the occipital bone. The double blade was placed ventrally and the single blade dorsally, according to the manufacturer’s recommendations [47]. Larger birds were restrained using large bags with head holes for operator safety during the clonic convulsion phase. CD was applied by a trained operator following the AVMA recommendations and Canadian poultry code guidelines for poultry euthanasia [2, 3]. Following euthanasia method application, birds were placed on a table in a sternal recumbent position and manually restrained by the legs to minimize EEG artifact caused by movement. A non-penetrating captive bolt device was applied to all animals 5 min following CD or MCD application. Birds were lightly anesthetized prior to euthanasia using a minimal anesthesia model protocol to ensure a humane death. Time to death was determined based on EEG responses and compared to behavioural and physiologic changes [16, 17]. Time recording started immediately after injection of barbiturate or as soon as the dislocation procedure had been completed.

### Electroencephalographic assessments

EEGs were recorded using a Trackit MK3 EEG Recorder with video (Lifelines Neurodiagnostic Systems, IL, USA). The EEG recording method was adapted from clinical techniques developed by a board certified veterinary neurologist (FJ). Impedance was kept under 16 kΩ in all cases. Electrode impedance was initiated prior to baseline recording and was recorded after completion of the euthanasia method. In addition, impedance was recorded if an electrode was not in place, primarily after significant clonic convulsions. Specific electrode input was later removed if the impedance was above 16 kΩ. Validation of EEG signals and technique was conducted using a blinded evaluation by a board certified veterinary neurologist unaware of euthanasia method, epoch timing, trial, age, or sex. Raw EEG recordings were reviewed manually for signal artifact and muscle activity prior to fast Fourier transformation (FFT) using 24 artifact-free 1 s epochs selected at 15 s intervals post euthanasia (20 × 1 s epochs obtained in a 5-min recording post euthanasia and 4 × 1 s epochs at baseline recording). All turkeys acted as their own control.

Quantitative EEG (QEEG) analyses of variables derived from power spectra (i.e. median frequency (F50), spectral edge frequency (F95) and total power (Ptot)) and frequency bands were conducted with Insight II software (Persyst Development Corporation, Prescott, AZ) using Hamming windows, with 256 points/window and 50% overlapping windows. A high-frequency (60 Hz) band filter was applied to eliminate noise based on Nevarez et al. (2014) [12]. Frequency bands were arranged into delta (1 to 4 Hz), theta (4 to 8 Hz), alpha (8 to 13 Hz), and beta (13 to 30 Hz). Increases in delta and theta patterns are correlated with states of insensibility and an isoelectric EEG reflects no brain activity or brain death in poultry [9, 24]. Similarly, insensibility in chickens and turkeys is described as occurring when there is an increase in the total power (Ptot) concurrently with decreases in F50 and F95 (i.e., frequencies below 50 and 95% of the total power) with death reported as an absolute decrease in Ptot values [7, 48]. All EEG recordings were randomized using a random number generator program (random.org) prior to being analyzed. The intraclass correlation coefficient (ICC) was calculated to assess accuracy of epoch selection prior to FFT (all intra-observer reliability tests produced an ICC above 0.8 in 10% of scored EEG recordings). Latency to isoelectric point was determined by visual inspection of the raw data (i.e., decreased wave amplitude with ≤2 uVpp and 2 consecutive isoelectric 1 s epochs) [49] by an observer blinded as to treatment.

### Physiologic and behavioural recording and scoring

Videos were recorded in front of and behind each bird using two high definition video cameras (JVC GZ-E200 full HD Everio Camcorder, Yokohama, Japan) mounted on tripods. Recording began prior to the application of treatment until five minutes after euthanasia method application. The response of each bird was scored continuously upon application of treatment by a single experienced observer blinded to treatment, trial and age using Observer XT program (Version 9.0: Noldus Information Technology, Wageningen, Netherlands) according to an ethogram designed for this study (Table 8). All video clips were assigned a random number as described. The intraclass correlation coefficient (ICC) was then determined to ensure parameters were scored consistently over time and no drift occurred (all intra-observer reliability tests produced an ICC above 0.75 in 20% of videos). Cardiac auscultation as well as presence of nictitating membrane and pupillary light reflexes were conducted every 60s until the five-minute endpoint to determine latency to cardiac arrest or latency to loss of brainstem reflexes, respectively. Cardiac arrest was measured by direct auscultation with a stethoscope in eight w.o. birds; due to the large body size and restraining technique it was not possible to estimate cardiac arrest in 18 w.o. birds. Reported results (Table 1) include incidence and percentage of physiologic and behavioural responses occurring in the immediate 5 min post method application application.

**Table 8:** Description of behavioural and physiologic parameters measured in turkeys using video recordings after euthanasia method application

Parameter	Description	Measures
Gasping	Repetitive and rapid opening and closing of the beak (not associated with respiratory function)	Latency Duration
Clonic convulsions	Rapid, uncoordinated movement of the body and wings	Latency Duration
Tonic convulsions	Slow extension or movement of the legs and wings	Latency Duration
Motionless	No body or respiratory movement	Duration
Feather erection	Multifocal to generalized rising of feather of the body	Latency Duration
Time to last body movement	Time of last wing, leg or body movement or onset of motionless with no further movement	Latency
Loss of nictitating membrane	Loss of movement of the third eyelid over the cornea after lightly touched with a fingertip	Latency
Loss of pupillary light reflex	Loss of constriction of the pupil after penlight was shone into the eye	Latency
Cardiac arrest	Cessation of function of the heart.	Latency

Adapted from Martin et al. (2016) [29].

Either latency, duration or both were recorded for each parameter.

### Statistical analyses

All statistical analyses were performed using SAS 9.4 (SAS Institute Inc., Cary, NC). EEG results were analyzed by use of linear mixed model that incorporated trial, age, euthanasia method and time as fixed effects, bird as a random effect and time as a repeated measure. The dependent variables were the EEG frequencies (alpha, beta, delta, theta, F50 and F95) and Ptot. The interactions between time and method and time × age × method were also included as a fixed effect. Only significant interactions between fixed effects are reported. Dunnett post hoc tests were conducted to identify group differences, relative to baseline, and a value of adjusted *p* < 0.05 was considered significant. A mixed model analysis was performed to test the fixed effect of age and euthanasia method and their interaction on the latency to isoelectric point.

A Fisher’s exact test was used to test the null hypothesis that the proportion of turkeys presenting reflex, behavioural, physiologic, and isoelectric EEG responses was independent of the euthanasia method. Frequency tables were used to determine the numbers of turkeys presenting each reflex and physiologic or behavioural parameter. Mixed model analyses were used to test fixed effects of the euthanasia method, trial, age and their interaction on the duration of and latency to clonic-tonic convulsions, gasping, and feather erection of birds presenting said parameters. Residuals plots were examined to check deviations from normality. The method × age interaction was removed from the model if the results showed no significant interaction and the data was pooled for both ages to test the euthanasia method and dependent value interaction. Post hoc tests were done on significant factors using the Tukey-Kramer adjustment (*p* = < 0.05). All variables raw means ± SE are reported in the results. Correlations between EEG and physiologic or behavioural measures of birds presenting an association were tested with a Pearson’s correlation test. A value of *p* < 0.05 was considered significant for all analyses.

## Additional files

Additional file 1: Mean (±SE) EEG parameters after administration of pentobarbital sodium to turkeys. (DOCX 16 kb)

Additional file 2: Mean (±SE) EEG parameters after manual cervical dislocation to turkeys. (DOCX 16 kb)

Additional file 3: Mean (±SE) EEG parameters after MCD application to turkeys. (DOCX 16 kb)

## Abbreviations

EEG: Electroencephalogram
F50: Median frequency
F95: Spectral edge frequency
Ptot: Total power
QEEG: Quantitative electroencephalogram
