# Supplementary material for: Electroencephalographic, physiologic and behavioural responses during cervical dislocation euthanasia in turkeys
Source: BMC Vet Res. 2019 May 7;15:132. doi: 10.1186/s12917-019-1885-x (PMC6505191; doi:10.1186/s12917-019-1885-x)
Supplement: Supplementary file 1 — Mean (±SE) EEG parameters after administration of pentobarbital sodium to turkeys. (DOCX 16 kb) [file 12917_2019_1885_MOESM1_ESM.docx]

**Additional file 1:** Mean (±SE) EEG parameters after administration of pentobarbital sodium to turkeys.

| Time (s) | Delta (μV) | Theta (μV) | Alpha (μV) | Beta (μV) | F50 (Hz) | F95 (Hz) | PTOT (μV) |
| --- | --- | --- | --- | --- | --- | --- | --- |
| Baseline | 1.8 (0.7) | 1.4 (0.3) | 0.7 (0.2) | 0.7 (0.2) | 5.1 (1.2) | 17.4 (1.8) | 2.3 (0.2) |
| 15 | 0.9 (0.7) | 0.7 (0.2)* | 0.5 (0.2) | 0.5 (0.2) | 5.7 (1.2) | 21.8 (1.8) | 1.2 (2.2)* |
| 30 | 0.3 (0.7) | 0.4 (0.2)* | 0.3 (0.2)* | 0.4 (0.2)* | 7.0 (1.2) | 25.6 (1.8)* | 0.7 (0.2)* |
| 45 | 0.4 (0.7) | 0.4 (0.2)* | 0.3 (0.2)* | 0.4 (0.2)* | 6.7 (1.2) | 24.8 (1.8)* | 0.8 (0.2)* |
| 60 | 0.4 (0.7) | 0.4 (0.2)* | 0.4 (0.2)* | 0.5 (0.2) | 9.7 (1.2)* | 25.3 (1.8)* | 0.9 (0.2)* |
| 75 | 0.4 (0.7) | 0.4 (0.2)* | 0.4 (0.2) | 0.5 (0.2) | 9.3 (1.2) | 24.0 (1.8)* | 0.8 (0.2)* |
| 90 | 0.4 (0.7) | 0.4 (0.2)* | 0.4 (0.2)* | 0.4 (0.2)* | 8.0 (1.2) | 24.1 (1.8)* | 0.8 (0.2)* |
| 105 | 0.4 (0.7) | 0.4 (0.2)* | 0.3 (0.2)* | 0.4 (0.2)* | 6.0 (1.2) | 24.6 (1.8)* | 0.7 (0.2)* |
| 120 | 0.3 (0.8) | 0.3 (0.3)* | 0.3 (0.2)* | 0.4 (0.2)* | 11.3 (1.2)* | 25.4 (1.8)* | 0.6 (0.2)* |
| 135 | 0.4 (0.7) | 0.4 (0.2)* | 0.3 (0.2)* | 0.4 (0.2)* | 5.7 (1.2) | 24.5 (1.8)* | 0.7 (0.2)* |
| 150 | 0.3 (0.7) | 0.3 (0.2)* | 0.3 (0.2)* | 0.4 (0.2)* | 6.3 (1.2) | 25.4 (1.8)* | 0.7 (0.2)* |
| 165 | 0.3 (0.7) | 0.3 (0.2)* | 0.3 (0.2)* | 0.4 (0.2)* | 6.7 (1.2) | 25.3 (1.8)* | 0.7 (0.2)* |
| 180 | 0.4 (0.7) | 0.4 (0.2)* | 0.3 (0.2)* | 0.4 (0.2)* | 6.3 (1.2) | 23.5 (1.8)* | 0.7 (0.2)* |
| 195 | 0.4 (0.7) | 0.4 (0.2)* | 0.3 (0.2)* | 0.4 (0.2)* | 6.3 (1.2) | 24.5 (1.8)* | 0.7 (0.2)* |
| 210 | 0.3 (0.8) | 0.4 (0.3)* | 0.3 (0.2)* | 0.4 (0.2)* | 7.4 (1.2) | 25.3 (1.9)* | 0.7 (0.2)* |
| 225 | 0.3 (0.8) | 0.4 (0.3)* | 0.3 (0.2)* | 0.4 (0.2)* | 6.0 (1.2) | 24.4 (1.9)* | 0.7 (0.2)* |
| 240 | 0.3 (0.8) | 0.4 (0.3)* | 0.3 (0.2)* | 0.4 (0.2)* | 6.0 (1.2) | 25.5 (2.0)* | 0.7 (0.2)* |
| 255 | 0.3 (0.9) | 0.4 (0.3)* | 0.3 (0.3)* | 0.4 (0.2) | 7.4 (1.3) | 23.8 (2.3)* | 0.7 (0.3)* |
| 270 | 0.2 (1.0) | 0.4 (0.3) | 0.3 (0.3) | 0.4 (0.2) | 7.7 (1.4) | 25.3 (2.5)* | 0.7 (0.3)* |
| 285 | 0.3 (1.1) | 0.5 (0.4)* | 0.3 (0.3)* | 0.3 (0.2) | 5.1 (1.5) | 24.2 (2.8)* | 0.8 (0.3)* |
| 300 | 0.5 (1.0) | 0.4 (0.4)* | 0.3 (0.3)* | 0.3 (0.2)* | 6.6 (1.6) | 24.6 (2.6)* | 0.8 (0.3)* |

Data are shown as mean values over consecutive 15s intervals after euthanasia, with baseline representing the mean of the 4s immediately prior euthanasia.

*indicate values within columns that are significantly different from baseline (adjusted *p* < 0.05)
