# Supplementary material for: Electroencephalographic, physiologic and behavioural responses during cervical dislocation euthanasia in turkeys
Source: BMC Vet Res. 2019 May 7;15:132. doi: 10.1186/s12917-019-1885-x (PMC6505191; doi:10.1186/s12917-019-1885-x)
Supplement: Supplementary file 2 — Mean (±SE) EEG parameters after manual cervical dislocation to turkeys. (DOCX 16 kb) [file 12917_2019_1885_MOESM2_ESM.docx]

**Additional file 2** Mean (±SE) EEG parameters after manual cervical dislocation to turkeys.

| Time (s) | Delta (μV) | Theta (μV) | Alpha (μV) | Beta (μV) | F50 (Hz) | F95 (Hz) | PTOT (μV) |
| --- | --- | --- | --- | --- | --- | --- | --- |
| Baseline | 4.1 (0.9) | 3.4 (0.2) | 1.4 (0.2) | 1.0 (0.2) | 3.9 (1.0) | 12.8 (2.1) | 5.1 (0.3) |
| 15 | 4.5 (0.9) | 3.8 (0.2) | 1.6 (0.2) | 1.1 (0.2) | 3.9 (1.0) | 11.7 (2.2) | 5.8 (0.3) |
| 30 | 4.1 (1.0) | 2.9 (0.3) | 1.4 (0.3) | 0.9 (0.2) | 3.6 (1.1) | 15.0 (2.3) | 4.8 (0.3) |
| 45 | 2.3 (0.9) | 2.0 (0.3) | 1.0 (0.2) | 0.8 (0.2) | 4.3 (1.1) | 14.9 (2.2) | 3.2 (0.3) |
| 60 | 4.5 (0.9) | 2.8 (0.3) | 1.3 (0.2) | 1.2 (0.2) | 5.8 (1.0) | 14.5 (2.1) | 4.9 (0.3) |
| 75 | 3.3 (1.0) | 2.4 (0.3) | 1.0 (0.3) | 0.8 (0.2) | 4.4 (1.1) | 14.2 (2.3) | 3.5 (0.3) |
| 90 | 1.6 (0.9) | 1.2 (0.3) | 0.4 (0.2) | 0.7 (0.2) | 4.8 (1.0) | 18.0 (2.1) | 2.1 (0.3) |
| 105 | 2.1 (1.0) | 1.8 (0.3) | 1.1 (0.2) | 1.0 (0.2) | 5.0 (1.1) | 17.9 (2.2) | 3.2 (0.3) |
| 120 | 1.2 (0.9) | 0.9 (0.3)* | 0.4 (0.2)* | 0.5 (0.2)* | 3.8 (1.0) | 19.0 (2.1) | 1.5 (0.3)* |
| 135 | 1.0 (0.9)* | 0.7 (0.3)* | 0.5 (0.2)* | 0.5 (0.2)* | 6.0 (1.0) | 21.3 (2.1)* | 1.3 (0.3)* |
| 150 | 0.7 (0.9)* | 0.5 (0.3)* | 0.4 (0.2)* | 0.5 (0.2)* | 8.5 (1.0) | 22.8 (2.1)* | 1.0 (0.3)* |
| 165 | 0.5 (0.9)* | 0.5 (0.3)* | 0.4 (0.2)* | 0.5 (0.2)* | 8.2 (1.1) | 23.4 (2.2)* | 0.9 (0.3)* |
| 180 | 0.8 (0.9)* | 0.6 (0.3)* | 0.4 (0.2)* | 0.5 (0.2) | 5.5 (1.0) | 23.8 (2.1)* | 1.1 (0.3)* |
| 195 | 0.6 (0.9)* | 0.5 (0.3)* | 0.4 (0.2)* | 0.5 (0.2)* | 6.4 (1.1) | 23.7 (2.2)* | 1.0 (0.3)* |
| 210 | 0.7 (0.9)* | 0.5 (0.3)* | 0.4 (0.2)* | 0.5 (0.2) | 6.8 (1.1) | 24.2 (2.2)* | 1.0 (0.3)* |
| 225 | 1.5 (0.9) | 0.8 (0.3)* | 0.5 (0.2)* | 0.6 (0.2) | 7.3 (1.0) | 22.0 (2.1)* | 1.4 (0.3)* |
| 240 | 0.8 (0.9)* | 0.5 (0.3)* | 0.4 (0.2)* | 0.4 (0.2)* | 8.5 (1.0) | 21.0 (2.1)* | 1.0 (0.3)* |
| 255 | 0.7 (0.9)* | 0.5 (0.3)* | 0.4 (0.3)* | 0.5 (0.2) | 6.6 (1.1) | 23.2 (2.3)* | 1.1 (0.3)* |
| 270 | 0.4 (1.0)* | 0.5 (0.3) | 0.5 (0.3)* | 0.6 (0.2) | 9.0 (1.2)* | 22.8 (2.5)* | 1.0 (0.3)* |
| 285 | 0.3 (1.0)* | 0.4 (0.3)* | 0.4 (0.3)* | 0.4 (0.2)* | 8.3 (1.1)* | 24.4 (2.4)* | 0.8 (0.3)* |
| 300 | 1.2 (1.0) | 0.4 (0.3)* | 0.4 (0.3)* | 0.4 (0.2)* | 6.9 (1.2) | 23.0 (2.4)* | 0.9 (0.3)* |

Data are shown as mean values over consecutive 15s intervals after euthanasia, with baseline representing the mean of the 4s immediately prior euthanasia.

*indicate values within columns that are significantly different from baseline (adjusted *p* < 0.05).
