# Supplementary material for: Electroencephalographic, physiologic and behavioural responses during cervical dislocation euthanasia in turkeys
Source: BMC Vet Res. 2019 May 7;15:132. doi: 10.1186/s12917-019-1885-x (PMC6505191; doi:10.1186/s12917-019-1885-x)
Supplement: Supplementary file 3 — Mean (±SE) EEG parameters after MCD application to turkeys. (DOCX 16 kb) [file 12917_2019_1885_MOESM3_ESM.docx]

**Additional file 3** Mean (±SE) EEG parameters after MCD application to turkeys.

| Time (s) | Delta (μV) | Theta (μV) | Alpha (μV) | Beta (μV) | F50 (Hz) | F95 (Hz) | PTOT (μV) |
| --- | --- | --- | --- | --- | --- | --- | --- |
| Baseline | 2.3 (0.5) | 1.9 (0.2) | 1.0 (0.1) | 0.8 (0.1) | 4.3 (0.8) | 15.9 (1.3) | 3.0 (0.2.) |
| 15 | 6.5 (0.6)* | 4.2 (0.2) * | 1.7 (0.2)* | 1.3 (0.1)* | 3.8 (0.9) | 12.3 (1.4) | 6.6 (0.2)* |
| 30 | 4.0 (0.6) | 2.6 (0.2) | 1.1 (0.2) | 0.9 (0.1) | 3.5 (0.8) | 13.3 (1.4) | 4.3 (0.2) |
| 45 | 3.6 (0.6) | 2.6 (0.2) | 1.2 (0.2) | 1.1 (0.1) | 3.7 (0.9) | 14.8 (1.4) | 4.2 (0.2) |
| 60 | 3.6 (0.5) | 2.6 (0.2) | 1.1 (0.2) | 0.9 (0.1) | 3.0 (0.8) | 14.5 (1.3) | 4.2 (0.2) |
| 75 | 2.8 (0.5) | 2.1 (0.2) | 1.0 (0.2) | 0.9 (0.1) | 3.8 (0.8) | 17.1 (1.3) | 3.3 (0.2) |
| 90 | 3.0 (0.6) | 1.9 (0.2) | 0.9 (0.2) | 0.8 (0.1) | 4.2 (0.8) | 16.4 (1.4) | 3.1 (0.2) |
| 105 | 3.5 (0.6) | 2.1 (0.2) | 1.0 (0.2) | 0.9 (0.1) | 3.6 (0.8) | 16.2 (1.3) | 3.4 (0.2) |
| 120 | 2.4 (0.5) | 1.6 (0.2) | 0.8 (0.2) | 0.8 (0.1) | 4.0 (0.8) | 17.1 (1.4) | 3.7 (0.2) |
| 135 | 2.2 (0.6) | 1.6 (0.2) | 0.8 (0.1) | 0.7 (0.1) | 4.2 (0.8) | 16.5 (1.3) | 2.7 (0.2) |
| 150 | 2.8 (0.5) | 1.5 (0.2) | 0.8 (0.1) | 0.8 (0.1) | 5.0 (0.8) | 17.3 (1.3) | 2.7 (0.2) |
| 165 | 2.5 (0.5) | 1.4 (0.2) | 0.7 (0.2) | 0.7 (0.1) | 4.8 (0.8) | 18.0 (1.3) | 2.5 (0.2) |
| 180 | 2.4 (0.6) | 1.6 (0.2) | 0.9 (0.2) | 0.8 (0.1) | 5.4 (0.8) | 18.2 (1.3) | 2.7 (0.2) |
| 195 | 2.0 (0.6) | 1.2 (0.2) | 0.6 (0.2) | 0.7 (0.1) | 6.0 (0.9) | 19.0 (1.4) | 2.1 (0.2) |
| 210 | 1.8 (0.6) | 1.2 (0.2) | 0.6 (0.2)* | 0.7 (0.1) | 4.6 (0.9) | 18.5 (1.3) | 2.0 (0.2) |
| 225 | 2.4 (0.5) | 1.1 (0.2) | 0.6 (0.2) | 0.7 (0.1) | 4.3 (0.9) | 19.6 (1.3) | 2.0 (0.2) |
| 240 | 3.0 (0.5) | 1.3 (0.2) | 0.6 (0.2)* | 0.6 (0.1) | 3.8 (0.9) | 17.5 (1.3) | 2.4 (0.2) |
| 255 | 1.9 (0.5) | 1.1 (0.2) | 0.6 (0.2)* | 0.6 (0.1) | 4.4 (0.9) | 18.6 (1.3) | 1.9 (0.2) |
| 270 | 2.1 (0.5) | 1.0 (0.2) | 0.5 (0.1)* | 0.6 (0.1) | 4.2 (0.8) | 19.7 (1.3) | 1.8 (0.2) |
| 285 | 2.0 (0.6) | 1.0 (0.2) | 0.6 (0.2)* | 0.6 (0.1) | 4.5 (0.9) | 12.8 (2.1) | 1.8 (0.2) |
| 300 | 1.3 (0.5) | 0.8 (0.2)* | 0.5 (0.2)* | 0.6 (0.1) | 4.4 (0.9) | 11.7 (2.2)* | 1.5 (0.2)* |

Data are shown as mean values over consecutive 15s intervals after euthanasia, with baseline representing the mean of the 4s immediately prior euthanasia.

*indicate values within columns that are significantly different from baseline (adjusted *p* < 0.05).
